# Encapsulation of Isoniazid-conjugated Phthalocyanine-In-Cyclodextrin-In-Liposomes Using Heating Method

**DOI:** 10.1038/s41598-019-47991-y

**Published:** 2019-08-07

**Authors:** Christian Isalomboto Nkanga, Rui Werner Maçedo Krause

**Affiliations:** Center for Chemico- and Bio-Medicinal Research (CCBR), Department of Chemistry, Rhodes University, PO Box 94, Grahamstown, 6140 Eastern Cape, South Africa

**Keywords:** Drug delivery, Nanoparticles

## Abstract

Liposomes are reputed colloidal vehicles that hold the promise for targeted delivery of anti-tubercular drugs (ATBDs) to alveolar macrophages that host Mycobacterium tuberculosis. However, the costly status of liposome technology, particularly due to the use of special manufacture equipment and expensive lipid materials, may preclude wider developments of therapeutic liposomes. In this study, we report efficient encapsulation of a complex system, consisting of isoniazid-hydrazone-phthalocyanine conjugate (Pc-INH) in gamma-cyclodextrin (*γ*-CD), in liposomes using crude soybean lecithin by means of a simple organic solvent-free method, heating method (HM). Inclusion complexation was performed in solution and solid-state, and evaluated using UV-Vis, magnetic circular dichroism, ^1^H NMR, diffusion ordered spectroscopy and FT-IR. The HM-liposomes afforded good encapsulation efficiency (71%) for such a large Pc-INH/*γ*-CD complex (PCD) system. The stability and properties of the PCD-HM-liposomes look encouraging; with particle size 240 nm and Zeta potential −57 mV that remained unchanged upon storage at 4 °C for 5 weeks. The release study performed in different pH media revealed controlled release profiles that went up to 100% at pH 4.4, from about 40% at pH 7.4. This makes PCD-liposomes a promising system for site-specific ATBD delivery, and a good example of simple liposomal encapsulation of large hydrophobic compounds.

## Introduction

Tuberculosis (TB) is a poverty related disease that represents the leading cause of mortality from microbial infections. The World Health Organization reported about 10.0 million of new TB cases with approximately 1.6 million deaths in 2017. Estimates of more than 90% of these deaths have been recorded in developing countries, where one TB patient dies every 15 seconds despite the existence of well-known anti-TB regimens^1^. The antimicrobial agents currently used for TB therapy are known as first-line anti-TB drugs (ATBDs), namely isoniazid (INH), rifampicin (RIF), pyrazinamide (PZM), ethambutol (ETMB); and second-line ATBDs such as ethionamide, streptomycin, ciprofloxacin and levofloxacin. While these ATBDs are still effective against drug-susceptible strains of the causative agent of TB, *Mycobacterium tuberculosis*^2^, the overall therapeutic success requires frequent administration of multiple drugs at high doses for 6–24 months^3^. Unfortunately, this is associated with various problems including severe adverse effects, poor patient adherence and drug resistance development^4^.

There are several reports that discuss the potential of colloidal drug delivery systems (CDDS) to improve the pharmacological profile and therapeutic outcomes of antimicrobials. The types of CDDS frequently used include liposomes, micelles, solid lipid micro- and nanoparticles, polymeric micro- and nanoparticles^2,5–8^. Amongst these, liposomes are phospholipid-based vesicular structures that are currently known as the most clinically used CDDS^9^. In the field of TB research particularly, liposomes have gained tremendous consideration as one of the most potential vehicles for ATBDs^2,4,6,10,11^. In addition, since *M. tuberculosis* is mostly located in macrophages^12^ that engulf liposomes by phagocytosis^13^, the use of pH-responsive liposomes holds the promise of site specific delivery of ATBDs in a controlled manner^14^. This approach is even more attractive if we consider the remarkable pH differences between the extracellular environments, mostly pH = 7.4, and the phagocytotic compartments, pH 4.5–6.5^15,16^.

**Figure 1:**
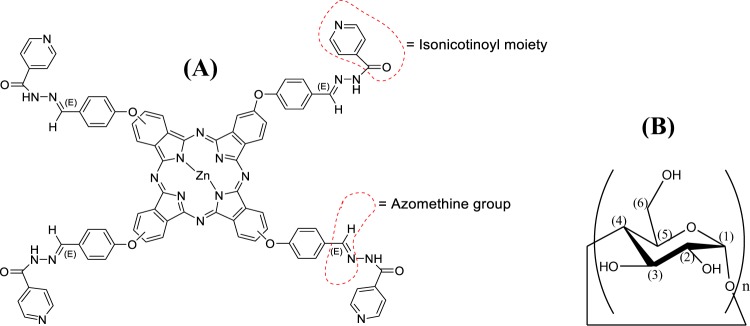
(**A**) Chemical structure of isoniazid grafted zinc (II) phthalocyanine (Pc-INH). **(B**) Chemical structure of α-D-glucopyranose unit, with n = 6–8 for natural CDs. The numbering (1) to (6) indicates the characteristic protons of CD molecules.

In this context, we looked at ways to deliver INH using an acid sensitive hydrolysable link that might as well be considered as a second tool for release monitoring, apart from being an antimicrobial agent with different mechanisms. Recently, we have considered covalent attachment of INH to hydrophobic fluorescent tags, namely zinc (II) phthalocyanines (Pcs), via hydrazone linkages to produce versatile hydrazone conjugates for liposomal controlled release (Pc-INH, Fig. 1A)^17^. This would further provide benefits for the development of novel multifunctional pharmacological or biochemical tools, while addressing the issues of liposomal delivery and drug leakage. In the case of INH, as with many other hydrophilic drugs, controlling the molecular size and other physical properties (e.g. solubility) of the pro-drug is a viable strategy for improving bioavailability^18^. Our preliminary data have provided proof of concept regarding pH-dependent release of INH from Pc-INH loaded liposomes^17^. However, Pc-INH has exhibited poor aqueous solubility that would be a concern in liposome technology. With the imperative use of organic solvents this concern can often be overcome, but usually at the expense of limited loading and encapsulation. We therefore further set ourselves the ambitious goal of low- or no-use of organic solvents without adversely affecting the encapsulation efficiency. Most reported Pcs currently in use as photosensitisers show a significant hydrophobic nature that limits their application and development. The case of Pc-INH is no different, hence the need to consider strategies to increase its solubility and allow further developments and direct pharmaceutical and biological applications^19–21^.

Pcs are excellent photosensitizers that have shown great potential for photodynamic therapy (PDT)^22,23^ and bio-imaging studies^24,25^. Several approaches have therefore been explored for improving their aqueous solubility, including molecular modifications, such as ring quaternization^26^, covalent conjugation to carbohydrates^27,28^, phosphonation or sulphonation^29,30^. Supramolecular approaches for complexation in hydrophilic macrocycles such as cucurbit[n]urils^20^ and cyclodextrins have also shown some success^29,31–33^.

Cyclodextrins (CDs) are naturally occurring macrocyclic oligosaccharides made of six, seven and eight α-d-glucopyranose units (Fig. 1B), which are known as *α*, *β* and *γ*-CD, respectively. Several CDs with GRAS status are commonly used as solubilising agents in pharmaceutical formulations as they comprise a large hydrophobic cavity that can host hydrophobic molecules (guest), and a hydrophilic outer surface that enhances guest aqueous solubility^34^. In 1994, McCormack and Gregoriadis made use of CD complexation to promote higher loading of hydrophobic compounds in liposomes, since the lipid bilayers provide a relatively limited space to accommodate sufficient amount of hydrophobic cargos^35^. Recently, many other groups have successfully encapsulated CD inclusion complexes in liposomes for controlled release purposes^36–42^. The advantages of liposomal encapsulation of drug-in-CD complexes, over conventional encapsulation of free drug in liposomes, include the possibility to achieve higher drug to lipid mass ratios, enhanced system stability and extended release of the cargoes^35,36^^,43^. Among numerous reports discussing drug-in-CD-in-liposomes, the work by Piel *et al*.^44^ illustrates well the positive impact of liposomal encapsulation of drug-in-CD complexes on formulation drug content. These authors obtained much better drug to lipid mass ratios when encapsulating betamethasone-in-CD than when loading betamethasone alone (as free drug). Another illustrative example of the benefits of CD inclusion complexes in liposome technology includes the work recently reported by Azzi *et al*.^45^. This group used the ethanol injection method to achieve liposomal encapsulation of the inclusion complex of hydroxypropyl-*β*-CD with a natural antimicrobial sesquiterpene (nerolidol). Results showed remarkable improvements in photostability and prolonged release of nerolidol from CD complex-loaded liposomes, in comparison with the conventional liposomes counterpart^45^.

In the present study, our hypothesis was that CD complexation would facilitate liposomal encapsulation of Pc-INH using an organic-solvent-free method, namely heating method, which is a simple, cost-effective and scalable preparation technique for liposomes^46^. We have prepared and fully characterized inclusion complex of *γ*-CD with Pc-INH, and achieved its liposomal encapsulation using a heating method. To the best of our knowledge, this is the first report on the formulation of Pc-in-CD-in-liposomes using a heating method. This is of paramount interest when considering the cost of liposome technology for possible up-scale production, which mostly requires special equipment such as microfluidic systems^47^ that may preclude wider development of therapeutic liposomes.

## Results and Discussion

### Phase solubility

**Figure 2:**
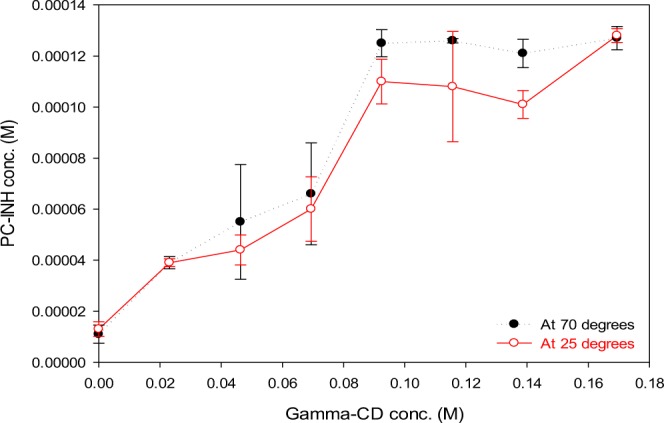
Phase solubility diagrams for *γ*-CD inclusion complexes with Pc-INH. The solubilizing effect of CD on Pc-INH in ultrapure water was noticeable, but not markedly affected by the change in experimental temperatures.

The phase solubility diagram of Pc-INH (as illustrated in Fig. 2 for *γ*-CD) showed B-type curves for all the CDs investigated, which suggests formation of poorly soluble complexes^48,49^. The aqueous solubility of Pc-INH at 25 °C (0.0016%) increased 4-, 8- and 11-fold with increasing amounts of *α-*CD (0–14%), *β-*CD (0–1.8%) and *γ*-CD (0–22%), respectively. The values for the apparent stability constant (*K*_*st*_) of the complexes were found to be in the following increasing order: *β-*CD (28.8548) < *α-*CD (67.3548) < *γ*-CD (86.8548), which is consistent with the aqueous solubility of respective native CD used. Although the *K*_*st*_ values obtained were below the optimal values, 100–1000 M^−1^, these data indicate the highest stability of *γ*-CD complexes. While complexation efficiency (*CE*) values for *α-*CD and *β-*CD were about 0.0007 and 0.0003, respectively, *γ*-CD’s *CE* was found to be 0.001, meaning at least one Pc-INH molecule is likely complexed by one in every 1000 molecules of *γ*-CD^48^. No significant differences were observed between the two experimental temperatures used.

### Complexation in solution

The formation of inclusion complexes between Pc-INH and *γ*-CD in dimethyl sulfoxide (DMSO) was verified by exploring the photophysical and photochemical properties of Pc-INH, such as UV-Vis absorption, magnetic circular dichroism (MCD), fluorescence quantum yields and lifetimes, and singlet oxygen quantum yields; along with the ^1^H NMR and DOSY profiles of *γ*-CD in deuterated DMSO (DMSO-d6).

**Figure 3:**
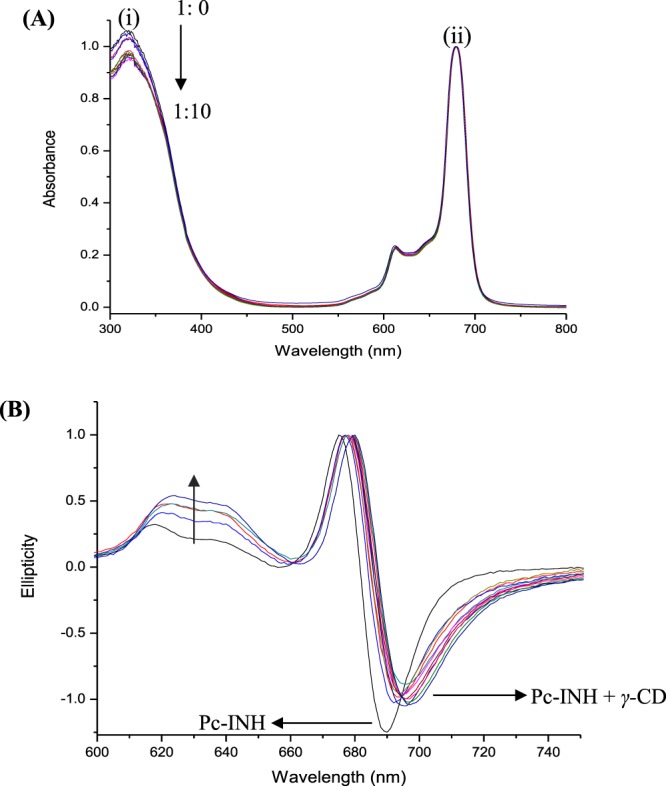
Effects of CD complexation on electronic transitions of Pc-INH in DMSO. **(A**) UV-Vis spectral changes for Pc-INH/*γ*-CD in different molar ratios (1:0 to 1:10), showing a reduction in the intensity of Pc-INH’s B-band (i) while its Q-band remains unchanged (ii). **(B**) Changes in MCD signals of Pc-INH in the presence of various amounts of *γ*-CD, with respective arrows indicating band shifts and increase in signals intensity on complexation.

Unlike Ogunsipe and Aletan^29^ who observed an increase in the Q-band intensity of a zinc phthalocyanine (ZnPc) upon CD complexation, the intensity of the B-band (at 320 nm) for Pc-INH was found to decrease with increasing amounts of CD as shown in Fig. 3A. Since the characteristic intense B band of Pc-INH arose upon conjugation of isoniazid (INH) to the aldehyde phthalocyanine^17^, the remarkable decrease in Pc-INH’s B-band intensity upon CD addition may be attributed to the inclusion of the isonicotinoyl moiety of Pc-INH in CD cavity. The change in the UV-Vis absorption spectra suggests complex formation since it indicates the degeneracy of the electronic structure of the guest’s chromophore, which is mostly due to guest interactions with CD’s inner groups or with solvent molecules being excluded from CD cavity^49,50^. Recently, Lu *et al*.^31^ have observed that changes in UV-Vis bands intensity of ZnPc were correlated with the guest-host molar ratio. Based on this observation, continuous variation of the UV-Vis absorption band (B band) intensity was considered in conjunction with Pc-INH to CD molar ratios (1:0 to 1:10) for estimation of Pc-INH:CD stoichiometry. Although all the CDs studied affected the UV-Vis spectra of Pc-INH, quite irregular spectral variations were observed with Pc-INH to CD molar interaction ratios for *α-* and *β-*CD (with 1:5 and 1:3 as respective optimal ratios). In comparison, *γ*-CD exhibited a much clearer relationship with an optimal ratio of 1:5. This molar ratio falls between 1:4 and 1:6 that were respectively reported by Lu *et al*.^31^ and Ogunsipe and Aletan^29^ for ZnPc/CD complexes. Since all the CDs increased the aqueous solubility of Pc-INH, the greater potential of *γ*-CD to markedly affect Pc-INH UV-Vis spectra may be due to its larger cavity size compared to *α-* and *β-*CD. This is consistent with the CD type-dependent complexation efficiency for ZnPc previously reported by Lu *et al*.^31^, and may be due to a kink configuration of the azomethine double bond in Pc-INH, which might have compromised inclusion in smaller CDs^51^.

MCD was employed as a specialized spectroscopic method that is complementary to UV-Vis absorption spectroscopy, to further verify changes in electronic structures of Pc-INH upon addition of *γ*-CD. As shown in Fig. 3B, the MCD spectrum of Pc-INH showed an S-like sigmoid curve at 600–700 nm with a crossover point at 682 nm, which approximates the λ_max_ observed in the UV-Vis spectrum of Pc-INH (681 nm). The MCD band around 682 nm was assigned to the Q-band of Pc-INH based on the Gouterman’s 4-orbital model, which suggests that the bands with high intensity are correlated to the changes in orbital angular momentum in the macrocyclic system^52^. Overall, the band feature in the MCD spectrum of Pc-INH corresponds to a pseudo-*A*_1_ term arising from the excited states that are orbitally degenerated^53^. While the Q-band of Pc-INH remained unchanged in UV-Vis spectra, distinctive spectral changes at the Q-band of Pc-INH was observed in the MCD spectra regardless of the amounts of *γ*-CD used (as pointed in Fig. 3B). This is in accordance with the optical theory, which stipulates that MCD can probe weak transitions that may not be perceived in conventional absorption spectra^54^. At lower wavelengths, a weaker MCD signal appeared at 300–400 nm (data not shown because of intense noise), and its intensity was found to increase for Pc-INH treated with *γ*-CD in the same way as observed at 600–720 nm. The remarkable differences in band intensity and position between the MCD signals of Pc-INH with and without *γ*-CD imply that the MCD does not have the same origin in the two cases^55^. This stands to reason, since the magnitude of MCD signal depends on the time-average of the total change in orbital momentum, which is closely related to a particular electronic transition and g-factor^56^. Therefore, the observed changes in MCD may indicate electronic perturbations in Pc-INH molecules, and serve as an evidence of successful inclusion complexation.

Similar to UV-Vis absorption spectra, the emission maxima wavelengths for Pc-INH in the presence and absence of *γ*-CD were found to be identical, 691 nm. However, enhancements in the fluorescence quantum yields (***Φ***_*F*_) and lifetimes (***τ***_*F*_) were observed following complexation of Pc-INH molecules by *γ*-CD. The presence of *γ*-CD molecules in solution has increased the Pc-INH ***Φ***_*F*_ and ***τ***_*F*_ from 0.129 and 2.865 ns to 0.179 and 2.891 ns, respectively. In contrast to Ogunsipe and Aletan^29^, who observed an increase in singlet oxygen quantum yields of ZnPc in DMSO upon CD complexation, the singlet oxygen quantum yield of free Pc-INH was found to be greater (***Φ***_***Δ***_ = 0.837) than that of Pc-INH treated with *γ*-CD (***Φ***_***Δ***_ = 0.601). These differences in photophysical and photochemical properties are not significant (p-value > 0.05) but may be valuable when considered as evidence of electronic perturbations in Pc-INH molecules due to inclusion in *γ*-CD cavity. Furthermore, based on ***Φ***_***Δ***_ and ***Φ***_*F*_, free Pc-INH would be a potential candidate for photodynamic therapy, while its *γ*-CD complex counterpart would be more suitable for fluorescence related applications such as bio-imaging and cell uptake studies.

**Figure 4:**
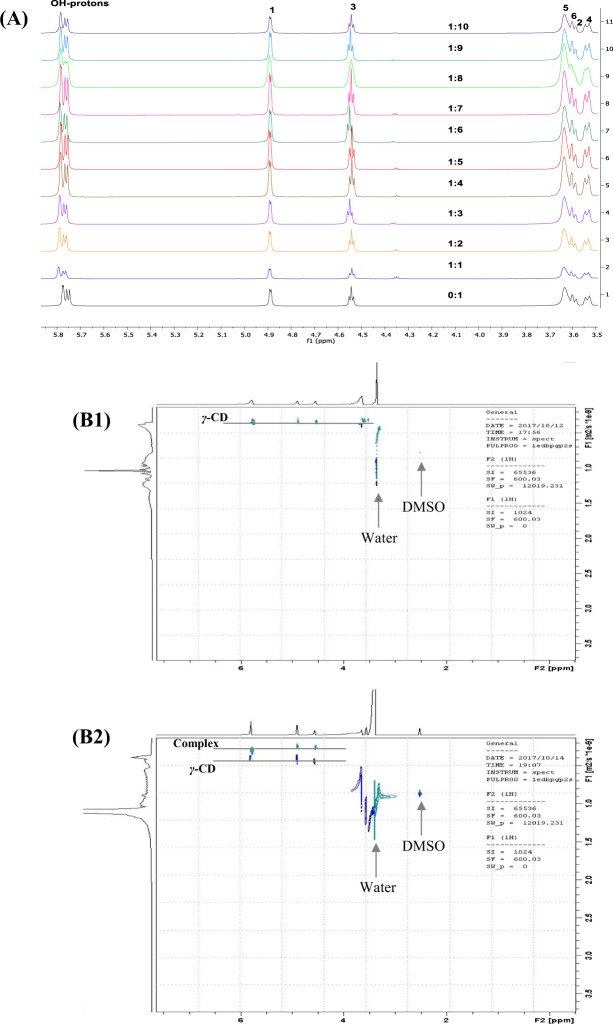
Effects of Pc-INH on the NMR profiles of *γ-*CD in DMSO-d6. **(A**) Partial ^1^H NMR spectra of *γ-*CD in different Pc-INH/γ-CD molar ratios (0:1 to 1:10), with numbers 1 to 6 indicating respective signals from *γ-*CD’s protons (1)–(6) (in reference with Fig. 1B). **(B1**) DOSY spectrum of *γ-*CD alone, exhibiting a set of proton signals aligned at the same diffusion coefficient (while the arrows indicate distinctive signals from the solvent). **(B2**) DOSY spectrum of *γ-*CD in the presence of Pc-INH (in Pc-INH/*γ-*CD molar ration of 1:2), showing the existence of two molecular species with distinctive diffusion coefficients: free and complexed *γ-*CD (complex).

Irrespective of the molar ratios used, the signals of the characteristic protons of *γ*-CD (labeled (1) to (6) in Fig. 1B) were found to be affected by the presence of Pc-INH molecules (Fig. 4A), with noticeable variations in ^1^H NMR chemical shifts (Δδ). In addition, the hydroxyl protons (OH-protons) of γ-CD appearing around 5.75–5.77 ppm also exhibited distinctive downfield shifts and marked peak broadening. Although no particular correlation was observed between the chemical shift’s changes and the molar ratios, the shifting of *γ*-CD peaks in the presence of Pc-INH is again a further valuable evidence of molecular interactions. Most importantly, the *γ*-CD protons on the internal wall of the cavity (H3 and H5), which appeared at 4.54 and 3.63 ppm, were found to be upfield- and downfield shifted, respectively, which may confirm formation of inclusion complexes between *γ*-CD and Pc-INH. Of particular interest, Δδ H3 was found to be ≤Δδ H5 for the samples containing 1:1, 1:2, 1:5, 1:7, 1:8 and 1:10 molar ratios, suggesting total inclusion of Pc-INH moiety in *γ*-CD cavity^57^.

As a useful NMR technique widely applied for analysis of mixtures, DOSY has demonstrated tremendous consideration in investigating supramolecular assemblies, including CD complexation as firstly studied by Lin *et al*.^58^. This is since CD complexes are characterized by greater molecular sizes and smaller *D* values compared to the native species^57^. In this effect, DOSY was used to provide evidence of complex formation between Pc-INH and *γ*-CD. Only data from samples containing Pc-INH and *γ*-CD in 1:2 molar ratio were considered for spectral analysis due to better signal-to-noise intensities compared to other samples with lower molar ratios. Figure 4B present the DOSY spectra for *γ*-CD in the presence and absence of Pc-INH.

The signals corresponding to *γ*-CD alone appear at 3.5–6 ppm with similar diffusion coefficient, about *D* = 37 × 10^*−11*^ *m*^2^*.s*^*−1*^. These signals are clearly far away from the signals related to DMSO (2.5 ppm) and water (3.3 ppm) as indicated in Fig. 4(B1). The trailing nature of the water signal is likely due to water molecules being partially trapped in the *γ*-CD cavity. In the presence of Pc-INH, *γ*-CD molecules exhibited two distinctive sets of signals with different diffusion coefficients (Fig. 4(B2)), *D* = 27 × 10^*−11*^ *and* 40 × 10^*−11*^ *m*^2^*.s*^*−1*^. In line with Stokes–Einstein equation^59^, the signals with greater *D* value can be allocated to free *γ*-CD while those with smaller *D* value may be assigned to complexed *γ*-CD molecules. Though *D* values for free *γ*-CD were not the same in the two cases as expected, the comparison of these spectra clearly shows the presence of new *γ*-CD species of different size^59^, thus indicating complex formation.

### Solid-state complexation

**Figure 5:**
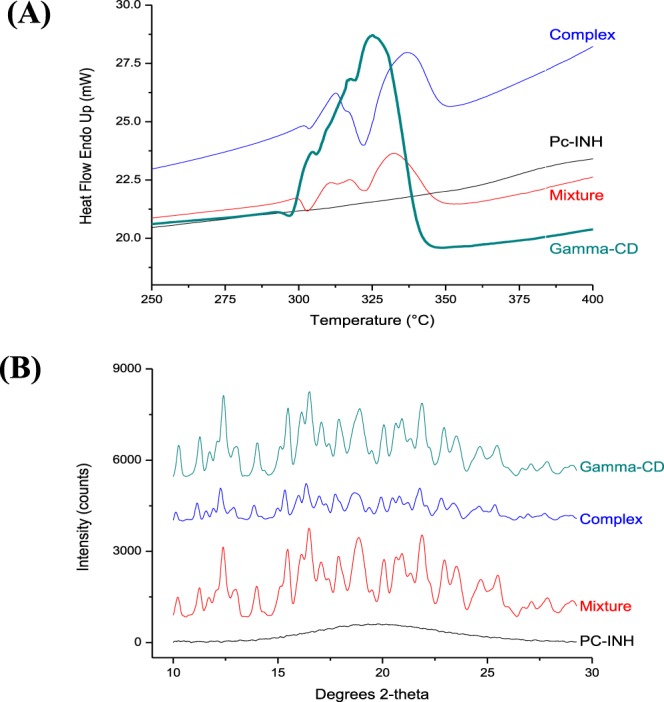
Effects of Pc-INH on the polymorphic form of *γ*-CD upon solid-state complexation. **(A**) Portions of DSC thermograms, depicting a change in the melting behavior of *γ*-*CD* in the presence of Pc-INH (appearance of new endotherms). **(B**) Portions of XRD diffractograms, showing broadening and intensity reduction of *γ*-CD’s crystalline peaks on complexation with Pc-INH.

Differential scanning calorimetry (DSC) is widely used to assess solid-state complexation with CD, since the characteristic peaks related to the thermal events of a guest may be shifted or disappeared when included in CD cavity^36,38,40^. Unfortunately, the present guest (Pc-INH) did not exhibit any clear peaks on DSC thermograms, despite several attempts. The DSC data for the ground products were, however, assessed in comparison with *γ*-CD. Pure *γ*-CD showed a sharp endothermic peak at 322.2 °C, while both physical mixture (mixture) and co-ground product (complex) exhibited two distinctive endotherms as shown in Fig. 5A. This may be indicative of strong interactions between *γ*-CD and Pc-INH arising from the different mixing processes employed (vortexing in the case of physical mixture and grinding for complex formation). Although these two materials showed similar DSC profiles, it turns out that the melting peaks from the complex have shifted to different extent compared to those from the mixture (Fig. 5A), suggesting existence of stronger guest-host interactions in the complex due to the grinding effect. This is somewhat consistent with data reported by Cavalcanti *et al*.^40^ who observed similar persistence and shifts of endothermic peaks of 2-hydroxypropyl-*β*-cyclodextrin in both its physical mixture and inclusion complex with β-lapachone.

As a powerful tool for solid-state analysis, X-ray diffraction (XRD) was used to assess formation of inclusion complex between Pc-INH and *γ*-CD. Figure 5B compares the portions of XRD patterns for raw materials, physical mixture and presumed complex. As expected based on DSC data, the XRD pattern of Pc-INH showed no crystalline peaks, whereas *γ*-CD’s pattern exhibited several sharp peaks that count for its crystalline nature. While the pattern of the mixture was an approximative superposition of that of raw *γ*-CD, the complex has exhibited a distinctive XRD profile with broader peaks of lower intensity. This is possibly related to variations in crystalline structure due to molecular interactions, since changes in both peak intensity and position indicate formation of new phase^60^. Although one can assume that the change in complex peak intensity is due to the grinding, since crystallites size reduction can affect the XRD pattern^61^, the agreement between our XRD and DSC data regarding potential molecular interactions suggests the presence of inclusion complex in the co-ground product^49^.

**Figure 6:**
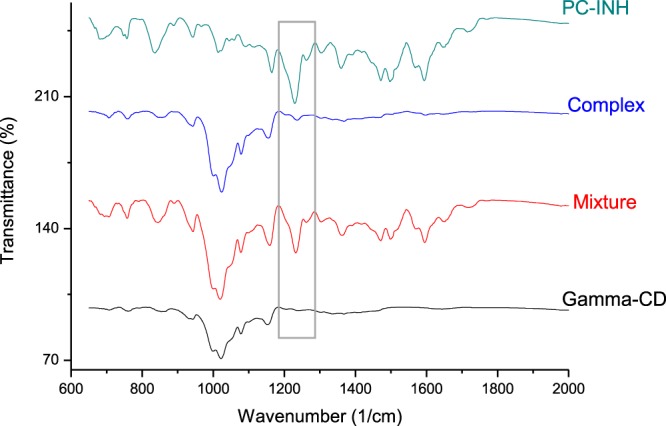
Partial FT-IR spectra, showing the effects of *γ*-*CD* on the stretching vibrations of Pc-INH on solid-state complexation, while the physical mixture (mixture) exhibits identical spectral features with raw Pc-INH. The vibration bands framed at 1235 cm^−1^ indicate the presence of Pc-INH in the solid-state complexation product (complex).

Since formation of intermolecular hydrogen bonding can cause significant changes in stretching vibrations^62^, FT-IR was considered as one of the techniques to assess solid-state inclusion of Pc-INH within *γ*-CD. Considering the FT-IR spectra of raw Pc-INH and *γ*-CD, the spectrum of physical mixture clearly corresponds to a simple combination of spectra of raw materials, which indicates lack of remarkable intermolecular interactions in the blend. However, only the most prominent band of Pc-INH, corresponding to C–O–C groups^31^, weakly appears at 1235 cm^−1^ in the FT-IR spectrum of the complex (as framed in Fig. 6); while all other characteristic bands are not noticeable. Noteworthy, such changes in relative intensities (including partial or total disappearance) of guest bands in FT-IR spectra of CD complexes have been observed before^40,50^, and can be explained by masking of the functional groups being included in CD cavity^49^. Considering the chemical structure of Pc-INH (Fig. 1A), the fact that the azomethine (CH=N) and carbonyl (C=O) stretches^17^ were not noticeable in the range of 1600–1800 cm^−1^ suggests that at least some of these groups are trapped inside CD cavity (leading to inapparent vibration bands). This observation is consistent with the UV-Vis spectral changes that were assigned to inclusion of some of the Pc-INH’s isonicotinoyl moieties in CD, which led to a decrease in Pc-INH’s B-band intensity (Fig. 3A).

**Figure 7:**
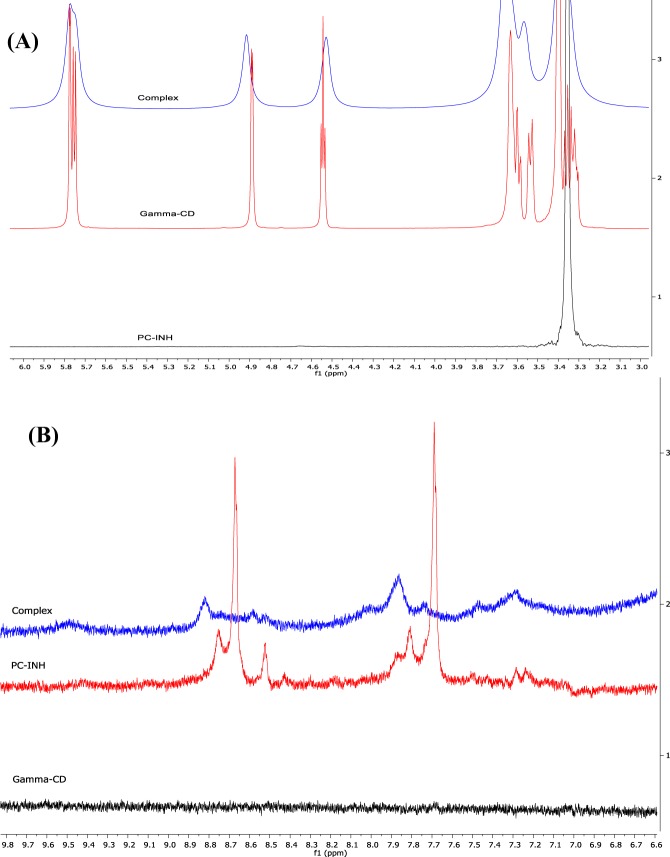
Effects of solid-state complexation on the ^1^H NMR profiles of *γ*-CD and Pc-INH, as co-ground product (complex) dissolved in DMSO-d6. **(A**) Comparison of spectral patterns of raw and co-ground *γ*-CD, exhibiting peaks broadening and shift that suggest existence of molecular interactions. **(B**) Comparison of spectral patterns of raw and co-ground Pc-INH, showing shift and disappearance of the resonance signals from Pc-INH’s aromatic protons.

The partial ^1^H NMR spectrum of the solid-state complex dissolved in DMSO-d6 was examined in comparison with the ^1^H NMR spectrum of each raw material (Fig. 7A). Much clear broadening and shift of *γ*-CD peaks can be observed when considering the complex ^1^H NMR profile against pure *γ*-CD counterpart. Comparison of the aromatic range of these spectra shows that, apart from the shift of peaks, some of the Pc-INH signals disappeared in the presence of *γ*-CD (Fig. 7B). This may be probably due to inclusion of Pc-INH in CD cavity and confirm the presence of some Pc-INH/*γ*-CD complex in the co-ground product.

In sum, variations in the XRD patterns of *γ*-CD (particularly peak broadening) and reduced relative intensities in the FT-IR bands of Pc-INH, along with the shift and disappearance of the peaks on ^1^H NMR spectra, suggest successful solid-state complexation of Pc-INH in *γ*-CD.

### Liposomal encapsulation of the complex

Next, we considered the encapsulation of the solid-state Pc-INH/*γ*-CD complex in crude soybean lecithin liposomes, followed by stimulated release studies. The encapsulation efficiency (%EE) of complex loaded liposomes are presented in Table 1. The %EE of the liposome formulations F1–F4 fell in the range of 58–70%. Despite the variations in hydrating conditions used for the heating method (HM), no significant differences were observed between the HM-liposomes prepared (*p*–value > 0.05). This suggests that ethylene glycol (EG) and propylene glycol (PG) could be potential alternatives to glycerol (GL) for preparation of HM-liposome by heating method (HM)^63^, and demonstrates the possibility for encapsulation of CD/drug complexes in HM-liposomes without hydrating agents. Interestingly, the hydrating agent-free formulation (F4) exhibited the highest %EE, about 71%. In comparison with the previous works on drug-in-CD-in-liposomes, this %EE appears slightly lower than the values reported by Chen *et al*.^37^ and Cavalcanti *et al*.^40^, but much better than those reported elsewhere^36,38,39^. As shown in Table 1, there is a significant difference between the %EE of F4 and that of its formulation counterpart prepared by thin film hydration method (*p*–value < 0.05). This enhances the cost-effectiveness of heating method in addition to its status of being an organic solvent free, simple and fast method^46^.

**Table 1:** Characteristics of liposomal formulations (*n* = 3).

Formulation code	PS ± SD (nm)	PDI ± SD	ZP ± SD (mV)	EE ± SD (%)
F1	398.2 ± 10.0	0.29 ± 0.03	−57.2 ± 1.27	64.24 ± 2.65
F2	245.7 ± 0.9	0.26 ± 0.02	−55.4 ± 1.21	58.91 ± 7.16
F3	267.1 ± 3.5	0.28 ± 0.04	−55.7 ± 1.36	63.82 ± 1.58
F4	239.7 ± 1.9	0.27 ± 0.02	−56.8 ± 0.86	70.68 ± 6.44
PM-liposomes	330.3 ± 9.3	0.29 ± 0.02	−59.8 ± 1.67	56.75 ± 1.67
Pc-liposomes	121.9 ± 1.12	0.25 ± 0.01	−55.8 ± 2.05	35.55 ± 3.61
FHM-liposomes	144.8 ± 0.7	0.25 ± 0.04	−52.7 ± 1.20	56.12 ± 3.96
Blank liposomes	652.7 ± 8.1	0.32 ± 0.01	−58.4 ± 0.38	NA

FHM: film hydration method; Pc: Pc-INH; PM: physical mixture. NA: not applicable. PS: particle size. SD: standard deviation. PDI: polydispersity index. ZP: Zeta potential. EE: encapsulation efficiency.

Most importantly, the %EE of liposomes F4 was found to be statistically greater than those exhibited by both liposomes prepared with physical mixture (PM) and Pc-INH alone. This can serve as an additional evidence for successful formation of Pc-INH/*γ*-CD inclusion complex by grinding method. Arguably, the poorest %EE of Pc-INH loaded liposomes (Pc-liposomes) may be related to the absence of *γ*-CD that would have increased Pc-INH solubility. Whereas limited %EE of PM-liposomes could be explained by possible competition between Pc-INH and lipid components of soybean lecithin for *γ*-CD cavity, which might have left most of the Pc-INH molecules loose due to the substantial amount of soybean lecithin used and strong interactions between lipid molecules and CDs^51^.

### Particle size and Zeta potential

The liposomes prepared exhibited average particle sizes in the range of 150–650 nm with PDI < 0.35 (Table 1), indicating acceptable homogeneity^40^. The complex-loaded liposome formulation F4 (hydrating agent-free formulation) and its counterpart composed of physical mixture exhibited remarkably larger size compared to liposomes made of Pc-INH alone. This is consistent with some of the previous data where drug-in-CD-in-liposomes exhibited bigger sizes than drug loaded liposomes^35,36^, but disagrees with other authors who reported contradictory results^38,41^. Surprisingly, the mean size of blank liposomes was found to be about 2.7-fold bigger than that of liposome F4 counterpart. This was also observed when comparing the size of complex-loaded liposomes prepared by film hydration method (FHM-liposomes) with that of blank liposomes prepared using the same method, 144.8 and 546.8 nm respectively. A possible explanation could be the fact that some of the Pc-INH/*γ*-CD complex disrupts the lipid bilayers to the cost of formation of larger vesicle. The difference in sizes between liposomes prepared by HM and those from FHM was expected on the basis of the literature information^35^.

As with our previous observations on soybean lecithin liposomes^64^, Zeta potential measurement revealed that the liposomes prepared have high negative surface charges (Table 1). Similar Zeta potential values were observed for all formulations despite the differences in hydration conditions, nature of cargo or preparation methods. This agrees with previous reports discussing the insignificant impact of CD on liposome Zeta potential^36,40^ and makes sense in this case since all these liposomes were of identical lipid composition (being all composed of crude soybean lecithin).

### Particle morphology

**Figure 8:**
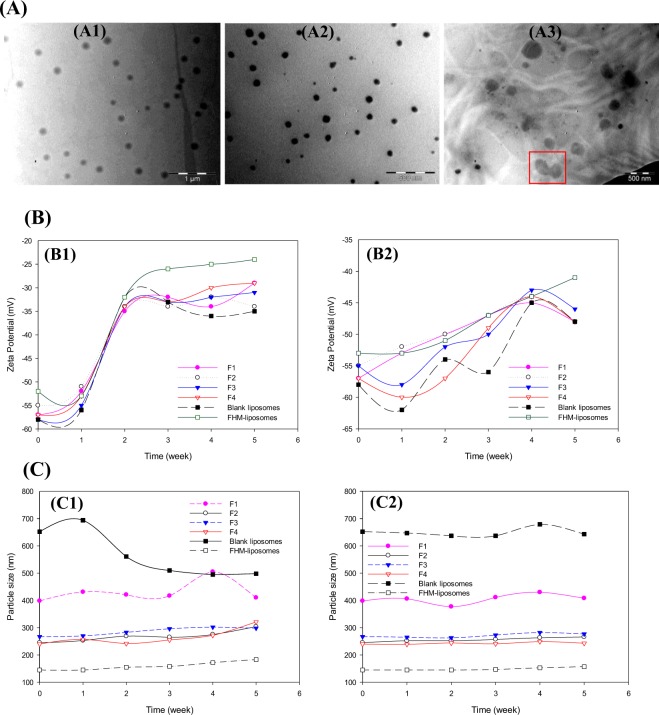
Particulate characteristics of liposomes under native and different storage conditions. **(A**) TEM micrographs of F4 freshly prepared (**A1**) and stored at 4 °C (**A2**) and rt (**A3**) for 5 weeks. **(B**) Comparing the variations in Zeta potentials of formulations stored at rt (**B1**) and 4 °C (**B2**). **(C**) Showing particles size stability at rt (**C1**) and 4 °C (**C2**) over 5 weeks of storage.

The microscopic analysis performed using transmission electron microscopy (TEM) revealed the presence of distinctive particles with spherical shape, which suggests successful formation of liposomal vehicles. The absence of particles aggregates maybe indicative of strong repulsive interactions between particles, probably due to their high surface charges as revealed by Zeta potential measurements. This makes the mean sizes determined by DLS trustworthy since this technique has been reported ineffective for samples that undergo particles aggregation^47^. Fig. 8A presents typical TEM micrographs for the freshly prepared formulation F4 and its aliquots stored over 5 weeks at 4 °C and room temperature (rt).

### System stability

Data from stability studies are presented in Fig. 8B,C. The studied formulations exhibited almost similar stability profiles regardless the presence or absence of the hydrating agent or Pc-INH/*γ*-CD inclusion complex. As shown in Fig. 8(B1), Zeta potential values (surface charge) of the liposomes stored at rt showed remarkable depletions after two weeks, followed by a steady state stability profile for the remainder, while those for the samples stored at 4 °C varied only slightly and irregularly over 5 weeks (Fig. 8(B2)). Interestingly, despite the variations in Zeta potential observed, the particle sizes remained almost unchanged throughout the test period irrespective of storage conditions (Fig. 8(C1),(C2)). Since surface charge governs the repulsive forces between particles and ensures formulation stability^47^, one can assume that the observed decrease in Zeta potential was just not substantial to favor noticeable particle clumping (which would have affected particles size). In other words, the obtained residual surface charge (e.g. −25 mV for FHM-liposomes stored at rt) seems to be efficient enough to minimize particles aggregation. Our observation is in accordance with data reported by Wang *et al*.^36^ who have studied the stability of drug-in-CD-in-liposomes over 16 days at 4 °C. As expected for samples stored at 4 °C, the microscopic analysis conducted before and after stability studies revealed the presence of individually well-dispersed and spherical particles (Fig. 8(A1),(A2)). Contrary to this, some particles fusion feature was observed in the TEM micrographs from rt samples as framed in Fig. 8(A3), supporting the marked variations in Zeta potential observed. This indicates much poorer stability of liposomes at rt compared to 4 °C, confirming the observation that particle collision and fusion are temperature dependent phenomena^65^.

### Surface elemental composition

**Figure 9:**
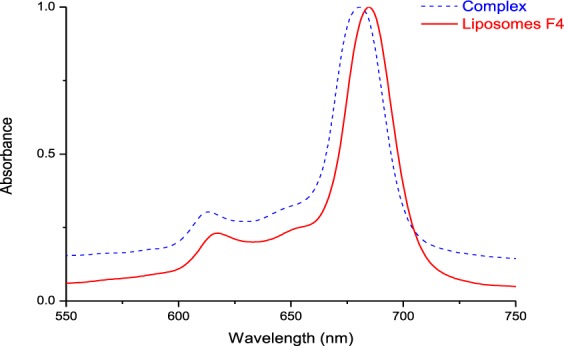
Comparison of the UV-Vis absorption spectra of Pc-INH/*γ*-CD complex (complex) and complex-loaded liposomes (liposomes F4) dissolved in DMSO after EDX experiments, confirming the presence of Pc-INH in the solid samples investigated by EDX.

Energy dispersive X-ray spectroscopy (EDX) experiments were performed in order to assess the surface elemental composition of the liposomal samples. While EDX data for Pc-INH/*γ*-CD complex revealed the presence of C, O and Zn; liposome F4 samples were found to be only made of C, O, P and Na (originating from the lecithin), and showed almost identical composition profiles with empty liposomes. This is consistent with our previous observation on Pc-INH loaded liposomses^17^, and questions the presence of Pc-INH molecules in the liposomes prepared. Although this is contradictory to the recent data that suggested possible adsorption of drug/CD complexes on liposomes surface^36^, the present EDX analysis coincides with the fact that the mean sizes of complex-loaded liposomes were not larger than that of empty liposomes and there was only a slight difference in their Zeta potential values. This suggests that Pc-INH/*γ*-CD inclusion complex has been embedded within lipid bilayers with higher affinity compared to aqueous phase, which may be correlated to the small value of stability constants (*K*_*st*_) observed during the phase solubility studies. The presence of Pc-INH in EDX liposomal samples was confirmed by UV-Vis spectroscopy in DMSO. The result showed a slight redshift in the Q-band region in comparison with the spectrum of the raw Pc-INH/*γ*-CD complex (Fig. 9), indicating existence of some molecular interactions between lipid components and Pc-INH. The B-band region (at 300–350 nm) was difficult to explore due to the potential interference observed with the UV absorption from the components of soybean lecithin (data not shown).

### pH-Dependent release profiles

**Figure 10:**
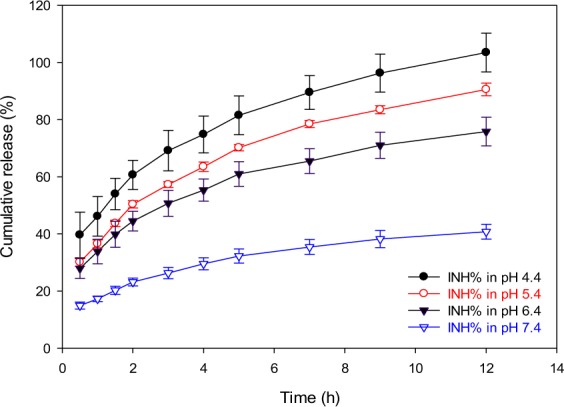
Release profiles of INH from Pc-INH/*γ*-CD complex-loaded liposomes F4. The study was conducted in citrate buffers (pH 4.4 and 5.4) and phosphate buffers (pH 6.4 and 7.4), showing that the amounts of INH released (INH%) depends on the acidity of release medium.

Data from *in vitro* release studies for Pc-INH/*γ*-CD complex-loaded liposomes (F4) conducted in media of different pH are presented in Fig. 10. The release kinetics of INH in neutral medium (pH 7.4) appeared to be much slower, with about 39% release over 12 hours, compared to those data observed in acidic conditions; where approximately 75, 90 and 100% release was observed at pH 6.4; 5.4 and 4.4, respectively. The pH-dependent release profile for complex loaded HM-liposomes seems to be similar to the release behavior exhibited by our previously reported Pc-INH loaded liposomes, except for pH = 7.4 where the present release percentage represents almost the double of the previous one, which was about 22%^17^. This may be attributed to inclusion of isonicotinoyl moiety of Pc-INH in *γ*-CD cavity, resulting in strong interactions that affect hydrolysis of the hydrazone moiety, which depends upon both hydroxide ions and protons^66^. Nevertheless, the observed pH-dependent release from the liposomes F4 encourages future investigations to evaluate potential controlled delivery of INH to alveolar macrophages following pulmonary administration^14,15,67^, since the lung lining fluid is known to be neutral while the endocytic compartments are naturally acidic^15^.

The release profiles obtained in different pH media were comparatively evaluated using the model dependent mathematical analysis of the amount of INH released to predict the release mechanisms and kinetics^68,69^. The release data were fitted into various mathematical models using the statistical software *DDSolver*^70^. The results from the release data modeling are presented in Table 2. Based on the coefficients of determination (R^2^), the following ranking orders can be considered for evaluation of the mathematical models explored for all the release media: Korsmeyer-Peppas > Baker-Lonsdale > First-order > Higuchi > Hixson-Crowell > Zero-order. Korsemeyer-Peppas’ model was found to best fit the release kinetics of INH from the prepared liposomes, as this model produced the highest R^2^ values irrespective of the release media^69^. This suggests that INH release kinetics could be likely influenced by erosion and/or diffusion^71^. Since the values of diffusion exponent (*n*) were found to be <0.45, it can be concluded that the transport mechanism of INH from the liposomes following hydrazone hydrolysis corresponds to a Fickian diffusion mode^72,73^.

**Table 2:** Data from model dependent mathematical analysis of INH release profiles

Model designation	Parameter	pH of the release medium			
		4.4	5.4	6.4	7.4
Zero-order	R^2^ Observed	−1.1654	−0.5274	−1.0925	−0.7970
	R^2^ Adjusted	−1.1655	−0.5274	−1.0925	−0.7970
First-order	R^2^ Observed	0.8271	0.7938	0.3866	−0.1928
	R^2^ Adjusted	0.8271	0.7938	0.3866	−0.1928
Higuchi	R^2^ Observed	0.7364	0.8673	0.7510	0.8137
	R^2^ Adjusted	0.7364	−0.5274	0.7510	0.8137
Korsmeyer-Peppas	R^2^ Observed	0.9968	0.9949	0.9950	0.9940
	R^2^ Adjusted	0.9964	0.9943	0.9944	0.9932
	*n*	0.3119	0.3518	0.3157	0.3331
Hixson-Crowell	R^2^ Observed	0.6991	0.6439	0.0846	−0.3756
	R^2^ Adjusted	0.6991	0.6439	0.0846	−0.3756
Baker-Lonsdale	R^2^ Observed	0.9812	0.9921	0.9308	0.8844
	R^2^ Adjusted	0.9812	0.9922	0.9308	0.8844

R:^2^ coefficient of determination. n: diffusion exponent.

## Conclusion

The present work reports successful encapsulation of an inclusion complex of *γ*-CD with Pc-INH in crude soybean lecithin liposomes using the heating method. The inclusion complexation was performed in solution and solid-state, and further extensively investigated using various spectroscopic techniques. The prepared liposomes exhibited good encapsulation efficiency and attractive pH-dependent release behaviour for possible site-specific delivery, as well as acceptable system stability under actual experimental conditions. However, since CD solubilizing effects on Pc-INH were not optimal (with stability constants < 100 M^−1^), this work introduces CD complexation as a strategic pre-treatment for organic solvent-free liposomal encapsulation of hydrophobic compounds, rather than a solubilisation approach commonly exploited when preparing drug-in-CD-in-liposomes. To the best of our knowledge, the present report appears to be the first of its kind discussing the application of this concept to phthalocyanines as versatile hydrophobic molecules, gathering both pH-responsive and fluorescent properties. Combining the cost-effectiveness of crude soybean lecithin^64^ and simplicity and scalability of heating method^46,74^, the present study looks inspirational for liposomal delivery and encapsulation of large/complex molecules as useful tools for pharmacological and biochemical applications, while still maintaining the inexpensive concepts. In line with drug delivery applications, this approach would be well-suited for development of multifunctional liposomal systems addressing poverty related diseases like TB. Nevertheless, the liposomes reported herein are made of a single anti-TB drug (i.e. isoniazid, derivatized as Pc-INH), while the use of monotherapy for TB is no longer recommended due to the alarming issue of antimicrobial resistance^75–79^. In this context, further studies exploring the possibilities to extend the therapeutic value of Pc-INH-in-CD-in-liposomes (by co-loading other anti-TB agents) are underway in our labs. Future investigations would include assessment of biocompatibility, intramacrophage targeted delivery and anti-tubercular activities.

## Experimental Section

### Chemicals

Dimethyl sulphoxide (DMSO), DMSO-d6, zinc (II) phthalocyanine (ZnPc), 1,3-diphenylisobenzofuran (DPBF), hydrochloric acid (HCl), citric acid, tri-sodium citrate, mono- and dibasic sodium phosphate, ethylene glycol (EG), propylene glycol (PG), glycerol (GL), *α*, *β* and *γ*-CD were sourced from Sigma Aldrich (Germany), or Wacker Chemie (Germany). Soybean lecithin was from Health Connection Wholefoods (USA). Methanol from Merck (Germany) and acetonitrile from Ranbaxy Fine Chemicals Ltd (India) were of analytical high-performance liquid chromatography (HPLC) grade. Isoniazid grafted zinc (II) phthalocyanine (Pc-INH) was synthesized and characterized as previously reported^17^.

### Equipment

Ground state electronic ultraviolet-visible (UV-Vis) absorption spectra were measured with a Shimadzu UV-2550 spectrophotometer. Magnetic circular dichroism (MCD) spectra were recorded on a Chirascan plus spectrodichrometer equipped with a 1 T (tesla) solid-state magnet using both the parallel and antiparallel fields. Fluorescence excitation and emission spectra were collected on a Varian Eclipse spectrofluorimeter. Fluorescence lifetimes were measured by means of a time-correlated single photon counting setup (TCSPC) (FluoTime 300, Picoquants GmbH) equipped with a diode laser (LDH-P-670, Picoquant GmbH, 20 MHz repetition rate, 44 ps pulse width). Irradiations for determination of singlet oxygen quantum yields were performed using a General Electric Quartz lamp (300 W), 600 nm glass (Schott) and water filters to filter off ultraviolet and far infrared radiations respectively. Proton nuclear magnetic resonance (^1^H NMR) and diffusion ordered spectroscopy (DOSY) experiments were conducted at room temperature (rt) using tetramethylsilane (TMS) as an internal reference on a Bruker AMX 600 MHz NMR spectrometer equipped with a pulse field gradient (PFG) probe. The pulse program was “ledbpgp2s” (longitudinal eddy current delay bipolar gradient pulse pair 2 spoil gradient). The diffusion delay was 200 ms and diffusion gradients were 1 ms sine-shaped pulses truncated at 0.5% of the top amplitude. The gradient strength was linearly incremented from 2 to 95% with 32 data points. The relaxation delay was 1 s and the diffusion time and gradient pulse length were 200 ms and 1 ms respectively. The number of scans was 32 and data were processed using Bruker TopSpin Software. The infrared (IR) spectra were recorded by the attenuated total reflection (ATR) method using a PerkinElmer Spectrum 100 FT-IR Spectrometer. Differential scanning calorimetry was performed from 25 to 445 °C using a PerkinElmer DSC-6000 instrument set at a flow rate of 10 °C/min in a nitrogen saturated atmosphere, and the inert nitrogen gas flow was 20 ml/min. X-ray diffraction (XRD) experiments were conducted on a Lynx Eye detector-equipped Bruker D8 Discover instrument using Cu-Kα radiation set at 1.5404 Å with a nickel filter, and the step size and slit width were 1°/min and 6.0 mm, respectively. A Zetasizer nano ZEN–3600 from Malvern Instruments was used for particle size and Zeta Potential determinations. Microscopic observations were performed on a Zeiss Libra–120 KV TEM instrument. An INCA PENTA FET connected to the VAGA TESCAM was used for energy dispersive X-ray spectroscopy (EDX) at 20 kV accelerating voltage. An Agilent HP1100 LC–MSD equipped with a quaternary pump, in-line degasser, DAD detector, 1100 MSD and ChemStation was used for HPLC analyses on a ZORBAX Elipse Plus C18 4.6 i.d.x 250 mm × 5 μm column. A Büchi Rotavapor R-205, MSE Mistral-1000, Digital Ultrasonic Cleaner/Spellbound-909, Deluxe Vortex Mixer/Chiltern MT19 and Apollo Scientific Lyo Lab-3000 were used for reduced pressure evaporation, centrifugation, sonication, vortex mixing and freeze-drying respectively.

### Phase solubility analysis

Five milligrams of Pc-INH were added to each 5 ml of CD aqueous solutions (0–14%; 0–1.8% and 0–22% for *α*, *β* and *γ*-CD, respectively), with a CD-free vial containing pure water as control. The resultant suspension was stirred over 24 hours at 25 °C. Two milliliters of the medium were withdrawn and filtered through a 0.45 micrometer syringe filter and evaluated by UV-Vis spectrometry for quantification of Pc-INH at 681 nm (λ_max_) using a freshly prepared calibration curve. On the other hand, 2 mg of Pc-INH was added to the remaining medium and the obtained mixture was stirred at 70 °C for 60 min. Upon cooling to room temperature (rt), the reaction medium was treated as described above for Pc-INH quantification. The phase solubility diagram was obtained by plotting Pc-INH molar concentrations against those of each CD. The straight line of the phase solubility diagram (which suggests a 1:1 complexation process) was considered for estimation of the apparent stability constant (*K*_*st*_) and complexation efficiency (*CE*) as previously reported by Hadžiabdić *et al*.^48^. This experiment was performed thrice for statistical purposes.

### Inclusion complexation studies

Various amounts of CD were added to each 8 ml of Pc-INH solution (0.1 mM) in DMSO to prepare multiple solutions containing different Pc-INH:CD molar ratios (1:0 to 1:10). These solutions were maintained at 70 °C under stirring over 24 hours. After cooling to rt, the medium was subjected to UV-Vis absorption and MCD spectrometric measurements at 300–800 nm. The continuous absorbance changes (ΔA) in UV-vis absorption spectra observed at 320 nm (B band of Pc-INH) were considered in conjunction with the molar interaction ratios between Pc-INH and CD (1:0 to 1:10) for stoichiometric evaluation^31^. Based on UV-Vis and phase solubility evaluations, only *γ*-CD was selected for further complexation studies. Following the same procedure, samples for ^1^H NMR and DOSY experiments were prepared in DMSO-d6. In this case, 500 µl of Pc-INH 7 µmol/ml (11 mg/ml) solution was mixed with various volumes (100–1000 µl) of *γ*-CD 3.5 µmol/ml (45 mg/ml) solution, and appropriate volumes of pure DMSO-d6 (900 – 0 µl) were added accordingly to afford a total volume of 1500 µl for each sample. These samples were evaluated in comparison with *γ*-CD solution as a control in order to explore possible changes in chemical shift values of *γ*-CD’s protons.

Further characterization addressed the photophysical and photochemical properties of Pc-INH in DMSO with and without *γ*-CD as described above. Fluorescence quantum yields (*Φ*_F_) were determined by comparative method using ZnPc standard as previously reported by Masilela *et al*.^30^. Fluorescence lifetimes (τF) were measured using a time-correlated single photon counting technique at rt as described by Segalla *et al*.^80^. Singlet molecular oxygen generation was evaluated in DMSO using the comparative method previously reported by Goslinski *et al*.^81^ with DPBF as a chemical quencher. Singlet oxygen quantum yield (*Φ*_Δ_) was calculated according to Yanık *et al*.^82^. All the experiments were repeated three times.

Solid state complexation was performed following the procedure reported by Ogunsipe and Aletan^29^ with slight modifications. Pc-INH and *γ*-CD were used in 1:5 molar ratio based on the stoichiometry estimated using the “continuous variation” method by means of UV-Vis spectroscopy. *γ*-CD (455 mg, 0.35 mmol) was moistened with distilled water to make a paste by kneading at rt. Afterwards, Pc-INH (110 mg, 0.07 mmol) was added portion wise under continuous milling over 30 min and the resulting paste was thoroughly milled for 60 min. Aliquots of the resultant product were dried to constant weight at 70 °C for 24 hours. A blend of Pc-INH and *γ*-CD (physical mixture) was vortexed for 1 min and dried as described above, alongside with separate materials to produce respective blanks. All the solid products prepared were comparatively analysed using DSC, XRD, FT-IR and ^1^H NMR in DMSO-d6 to confirm the presence of the inclusion complex prior to liposomal encapsulation.

### Liposomes preparation

Pc-INH/*γ*-CD complex from solid state experiment was encapsulated in liposomes using a heating method (HM) as described in the literature^46,74^ with a slight variation; involving 3 hydrating adjuvants, namely ethylene glycol (EG), propylene glycol (PG) and glycerol (GL) (Table 3). Soybean lecithin (100 mg) and the inclusion complex (25 mg) were hydrated with ultrapure water (10 ml) at rt for 60 min. Accordingly, a hydrating adjuvant (180 µl) was added to this mixture and the resultant medium was heated at 70 °C under stirring for 60 min. After cooling at rt, the volume of the preparation was adjusted to 15 ml with ultrapure water, and the mixture was low-speed centrifuged (LSC) at 4,000 rpm over 5 min for removal of non-encapsulated particles from liposomal suspension^83^. While this LSC-pellet was isolated for evaluation of encapsulation efficiency (EE, as detailed in the next section), the decanted liposomal supernatant was sonicated at 60 °C for 20 minutes. Aliquots (10 ml) of the obtained dispersion were subjected to high-speed centrifugation (HSC) for isolation of liposomes at 20,000 rpm on a Beckman Coulter Allegra 64 R set at 25 °C over 20 min. The obtained HSC-pellet was freeze-dried for further solid-state analysis, whereas the resultant HSC-supernatant was added to the previous LSC-pellet for EE evaluation. Based on the EE obtained, the HM-liposome formulation F4 was selected for further evaluations. Two F4 formulation counterparts containing the physical mixture (blend of Pc-INH and *γ*-CD in 1:5 molar ratio) and Pc-INH alone were prepared alongside with the corresponding blank HM-liposomes for comparison purposes.

**Table 3:** Composition of HM-liposome formulations.

Formulation code	F1	F2	F3	F4
**Hydrating adjuvant**	EG	PG	GL	*

^*^No hydrating adjuvant.

On the other hand, Pc-INH/*γ*-CD complex-loaded liposomes were prepared using film hydration method (FHM) as follows: the mixture of soybean lecithin (100 mg), inclusion complex (25 mg) and chloroform (2 ml) was dried at 40 °C using a rotary evaporator for 5 min. The round bottom flask was removed and stored in a desiccator overnight at rt. The obtained thin film was hydrated with 10 ml of ultrapure water at 70 °C under stirring for 60 min, and the resultant suspension was treated in the same way as described above to yield FHM-liposomes.

### **Determination of encapsulation efficiency (EE**)

DMSO (30 ml) was added to the LSC-pellet sample, and the resultant solution was subjected to UV-vis spectrometry at λ_max_ of Pc-INH (681 nm) for determination of EE^84^. This was performed in conjunction with lipid-free formulations (control) that were produced following the preparation procedure described above in order to get a theoretical total absorbance for Pc-INH at 681 nm in the actual experimental conditions. The %EE was calculated using the following equation^85^:

1 $$ \% EE=\frac{Theoretical\,total\,absorbance-LSC\,pellet\,absorbance\,}{Theoretical\,total\,absorbance}\times 100$$

### Spectroscopic and microscopic characterization

The particle size, size distribution and zeta potential of the sonicated liposome suspensions were determined by dynamic light scattering (DLS). Triplicate measurements were performed at rt under the scattering angle of 173°. Thereafter, samples were subjected to particle shape analysis using transmission electron microscopy (TEM). A drop of each DLS sample was placed on a copper grid and liquid in excess was absorbed with filter paper. The samples were allowed to dry at rt for 48 hours prior to TEM experiments. On the other hand, freeze-dried liposomal samples (from HSC-pellet) were evaluated for surface elemental composition using EDX in comparison with freeze-dried inclusion complex and blank liposomes. Further, samples (5 mg) from EDX experiments were dissolved in DMSO (2 ml) and the solution was subjected to UV-vis spectroscopy at 300–800 nm to confirm the presence of Pc-INH with regards to its characteristic absorption band in near infrared region, Q-band.

### Stability studies

All the loaded HM-liposomes (F1–F4) were subjected to stability studies in comparison with blank HM-liposomes and FHM-liposomes. Aliquots (2 ml) of each liposome suspension from sonication were stored in the dark at rt and 4 °C. Particle size and Zeta Potential measurements were performed on weekly basis over 5 weeks using DLS. Particle morphology was examined before and after stability studies by means of TEM.

### INH release studies

Prior to release studies, INH content in F4 was evaluated following the method previously reported^67^. Briefly, freeze-dried samples (25 mg) were incubated in ultrapure water (5 ml) at rt for 30 min. On one hand, the resultant dispersion (1 ml) was placed in a 10 ml volumetric flask containing methanol (4 ml). To this mixture, HCl 32% (3928 µl) was carefully poured. The mixture was sonicated for 30 min at 60 °C to ensure both liposomal disruption and cleavage of the hydrazone bonds to free INH molecules. After cooling to rt, the volume of the resultant suspension was adjusted to the mark with methanol prior to filtration using 0.22 µm syringe filters. The filtered solution was evaluated for INH concentration using an HPLC method previously reported^64^, with INH standard treated in the same conditions for establishment of the calibration curve. INH content (%C) was estimated according to the following equation^67^:

2 $$ \% C=\frac{\mathrm{Re}\mathrm{cov}ered\,INH\,amount}{Freeze\,dried\,Liposomes\,amount}\times 100$$

On the other hand, 1 ml of the above dispersion was used for INH release study using the dialysis method described by Nkanga *et al*.^64^ with slight modifications. Different media of pH (4.4–7.4) were used in order to evaluate the pH-dependent release of INH from F4 in conjunction with phagocytotic pathways^16^. The release study was conducted at 37 °C using citrate buffer solutions for pH 4.4 and 5.4, whereas phosphate buffers were used for pH 6.4 and 7.4 media. The dialysis tubing cellulose membrane used was Membra-Cel MD 10–14 × 100 CLR (Sigma Aldrich, Germany). At specific time intervals (0.5; 1; 1.5; 2; 3; 4; 5; 7; 9 and 12 hours), 5 ml of the release medium was withdrawn and equivalent volume of fresh buffer was added to maintain sink conditions. The concentration of INH in the release medium was evaluated by HPLC as previously reported^64^. All the experiments were conducted in triplicate. The release data from different pH media were comparatively evaluated using several mathematical models for estimation of the mechanisms and kinetics of release^68,69^. The release profiles were fitted into various mathematical models using the statistical software *DDSolver*, which is a menu-driven add-in programme for Microsoft Excel written in Visual Basic for modelling and comparison of drug dissolution profiles^70^.
